# Demonstration of full polarization control of soft X-ray pulses with Apple X undulators at SwissFEL using recoil ion momentum spectroscopy

**DOI:** 10.1107/S1600577524006325

**Published:** 2024-08-09

**Authors:** Christoph Kittel, Antoine Sarracini, Sven Augustin, Ningchen Yang, Andre Al Haddad, Eugenio Ferrari, Gregor Knopp, Jonas Knurr, Ana Sofia Morillo-Candas, Iwona Swiderska, Eduard Prat, Nicholas Sammut, Thomas Schmidt, Christoph Bostedt, Marco Calvi, Kirsten Schnorr

**Affiliations:** a Paul Scherrer Institut Forschungsstrasse 111 5232Villigen PSI Switzerland; b University of Malta MSD2080Msida Malta; c LUXS Laboratory for Ultrafast X-ray Sciences, Institute of Chemical Sciences and Engineering École Polytechnique Fédérale de Lausanne CH-1015Lausanne Switzerland; d Deutsches Elektronen-Synchrotron DESY Germany; RIKEN SPring-8 Center, Japan

**Keywords:** soft X-rays, FELs, polarization, undulator, COLTRIMS, Apple X, reaction microscope, recoil ion momentum spectroscopy

## Abstract

The ability to freely control the polarization of X-rays enables measurement techniques relying on circular or linear dichroism, which have become indispensable tools for characterizing the properties of chiral molecules or magnetic structures. Therefore, the demand for polarization control in X-ray free-electron lasers is increasing to enable polarization-sensitive dynamical studies on ultrafast time scales. The soft X-ray branch Athos of SwissFEL was designed with the aim of providing freely adjustable and arbitrary polarization by building its undulator solely from modules of the novel Apple X type. In this paper, the magnetic model of the linear inclined and circular Apple X polarization schemes are studied. The polarization is characterized by measuring the angular electron emission distributions of helium for various polarizations using cold target recoil ion momentum spectroscopy. The generation of fully linear polarized light of arbitrary angle, as well as elliptical polarizations of varying degree, are demonstrated.

## Abstract 2

The polarization capabilities of SwissFEL’s Athos beamline are demonstrated by deriving the Stokes parameters as a function of the undulator phase and comparing them with experimental measurements obtained by cold target recoil ion momentum spectroscopy.

## 1. Introduction

The ability to control the polarization of an X-ray beam opens up a wide field of scientific applications. Experimental techniques which rely on the linear or circular dichroism of molecules and materials have become indispensable tools in the field of X-ray spectroscopy at synchrotrons over the past decades (van der Laan *et al.*, 1986 ▸; Chen *et al.*, 1990 ▸; Ade & Hsiao, 1993 ▸; de Groot, 1994 ▸; Stöhr, 1995 ▸; Huang *et al.*, 2004 ▸; Stamm *et al.*, 2007 ▸; Wietstruk *et al.*, 2011 ▸; van der Laan & Figueroa, 2014 ▸). Such measurements require rotating the linear polarization from, for example, linear horizontal (LH) to linear vertical (LV) or changing the helicity from, for example, circular right (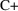) to circular left (C−). X-ray circular dichroism (XCD), for instance, is a powerful method for obtaining stereochemical information on molecules (Hergenhahn *et al.*, 2004 ▸), and X-ray magnetic circular dichroism (XMCD) is routinely used to study the properties of magnetic materials (Stöhr, 1995 ▸). Thus, the extension of such techniques into the femtosecond time domain using X-ray free-electron lasers (XFELs) represents a major step forward in studying polarization-dependent ultrafast processes (Higley *et al.*, 2016 ▸; Malvestuto *et al.*, 2022 ▸; Ilchen *et al.*, 2021 ▸; Yamamoto *et al.*, 2019 ▸; Rouxel & Mukamel, 2022 ▸).

XFELs are light sources which deliver photon beams with high brightness and short pulse duration of typically few to tens of femtoseconds to enable the investigation of ultrafast processes. The majority of XFEL facilities to date (Emma *et al.*, 2010 ▸; Ishikawa *et al.*, 2012 ▸; Kang *et al.*, 2017 ▸; Decking *et al.*, 2020 ▸; Prat *et al.*, 2020 ▸) employ planar undulators, which produce linearly polarized light with a fixed angle. This is mostly due to the simpler design of planar undulators compared with helical ones and especially so compared with undulators with variable polarizations.

Currently, two undulator-based methods exist to generate variable-polarized XFEL photon beams: (1) the so-called afterburner scheme, where a standard planar undulator line is followed by a short variable polarization undulator consisting of Apple (Clarke, 2004 ▸) or Delta (Nuhn *et al.*, 2015 ▸) type modules and (2) a complete undulator line consisting only of Apple or Delta type modules with full polarization control capabilities. The afterburner scheme was successfully demonstrated at LCLS in Stanford, USA (Lutman *et al.*, 2016 ▸), where a circular polarized X-ray beam with a few hundred microjoules was produced. The ongoing upgrade to LCLS-II (Raubenheimer, 2009 ▸; Tian & Nuhn, 2019 ▸) will also include an afterburner. Commissioning of an afterburner has begun on the SASE3 beamline of the European XFEL in Hamburg, Germany (Li *et al.*, 2017 ▸; Yakopov *et al.*, 2022 ▸). An afterburner design can be a more practical solution to add variable polarization to existing planar-polarized XFELs with enough available space for the additional installations, as most of the undulator line can remain to be of simpler and lower-cost planar undulator modules. A potential drawback is the generally lower attainable pulse energy for clean, variable polarizations, since most undulator modules do not contribute significantly in this scheme, while still adding towards a large footprint of the undulator line itself. Furthermore, the design requires the inclusion of an XFEL collimator. Full polarization control is implemented at FERMI in Trieste, Italy, in both its beamlines (FEL-1 and FEL-2) (Allaria *et al.*, 2015 ▸; Roussel *et al.*, 2017 ▸), using an undulator line which consists of six modules of the Apple II design, generating photons from the ultraviolet to the soft X-ray spectral range (up to around 300 eV). This design allowed also investigation of a simpler crossed polarized undulator scheme (Ferrari *et al.*, 2015 ▸). While full polarization control in the entire undulator line comes at the expense of requiring all undulator modules to be of a more complex and expensive type, it also offers several advantages: the undulator line is generally shorter, allowing a compact machine design, provides cleaner polarization without the need to suppress any unwanted contributions, and ensures that all modules contribute to the FEL lasing process resulting in higher pulse energies for all polarizations. In addition, experiments which do not require specific polarizations tend to gain from helical compared with standard planar polarization, since helically polarized undulators can offer a significantly shorter saturation length and higher saturation power, due to better coupling between the electron and the photon beam (Kittel *et al.*, 2024 ▸).

The soft X-ray branch Athos is the most recent addition to SwissFEL at the Paul Scherrer Institute in Villigen, Switzerland (Prat *et al.*, 2020 ▸). It is by design an extremely versatile undulator line offering full polarization control over the full Athos energy range of 0.25 keV to 1.8 keV. Its commissioning started in 2019 and it has been in user operation since 2021 (Prat *et al.*, 2023 ▸). Athos is the first beamline to employ Apple X undulator modules, the newest development in the well known Apple series: I, II and III (Calvi *et al.*, 2017 ▸). Due to their radial symmetry, Apple X type undulator modules have the additional capacity to access the full range of the undulator parameter *K* at all elliptical polarizations, without automatically generating a gradient. The undulator line consists of 16 Apple X modules with intra-undulator sections containing small magnetic chicanes between every two modules (Prat *et al.*, 2016 ▸), which also act as phase shifters. The undulator line is further split into two equal parts by a large magnetic chicane, offering a delay from −40 fs to 500 fs independent of polarization for two-color operation (Prat *et al.*, 2022 ▸). The flexible polarization control within the undulator line allows a split-undulator operation, in which different parts of the undulator can produce not only two pulses with different photon energies but also two different polarizations.

The polarization of soft X-rays can either be determined directly by diagnosing the properties of the generated light or indirectly by detecting secondary particles which were generated by the X-rays and thus carry information on the polarization. In polarimeters (Staub *et al.*, 2008 ▸; Allaria *et al.*, 2014 ▸), an analyzer crystal is typically rotated around the beam axis and differences in X-ray transmission or reflection are analyzed to retrieve the polarization state of the X-ray beam. An alternative common method to determine the polarization relies on measuring the well characterized angular emission patterns of photoelectrons ejected from rare-gas atoms by polarized light. One experimental realization to do so makes use of an assembly of electron spectrometers mounted around the beam propagation direction to measure the electron yield at different angles to reconstruct the polarization. This technique has been successfully applied at multiple facilities for polarization characterization (Viefhaus *et al.*, 2013 ▸; Veyrinas *et al.*, 2013 ▸; Allaria *et al.*, 2014 ▸; Lutman *et al.*, 2016 ▸; Hartmann *et al.*, 2016 ▸). Here, we employ cold target recoil ion momentum spectroscopy in order to reconstruct the angular electron emission pattern to characterize the variable X-ray polarizations generated by the Apple X undulators at Athos. Analogous to photoelectron spectroscopy, this measurement technique relies on the well characterized photoelectron emission patterns from polarized light. However, instead of detecting the photoelectrons at different angles directly, we detect the recoil ion momenta which are equal to the photoelectron momenta through momentum conservation. Using a so-called Reaction Microscope (ReMi) or Cold Target Recoil Ion Spectrometer (COLTRIMS) (Ullrich *et al.*, 2003 ▸), we are able to reconstruct the full three-dimensional electron emission pattern for each polarization. This is a clear advantage compared with measurements made with arrays of electron detectors, where covering all emission angles is almost impossible due to the amount of spectrometers that would be needed.

In the present work, the dependence of the X-ray polarization as a function of the undulator phase is derived for both linear and elliptical polarization modes, where the undulator phase is defined as the relative, longitudinal position of the magnetic arrays of the undulator module. This model is then compared with experimental measurements of the polarization state accomplished using recoil ion momentum spectroscopy of photoionized He gas.

## 2. Design of the Apple X undulator

**Figure 1:**
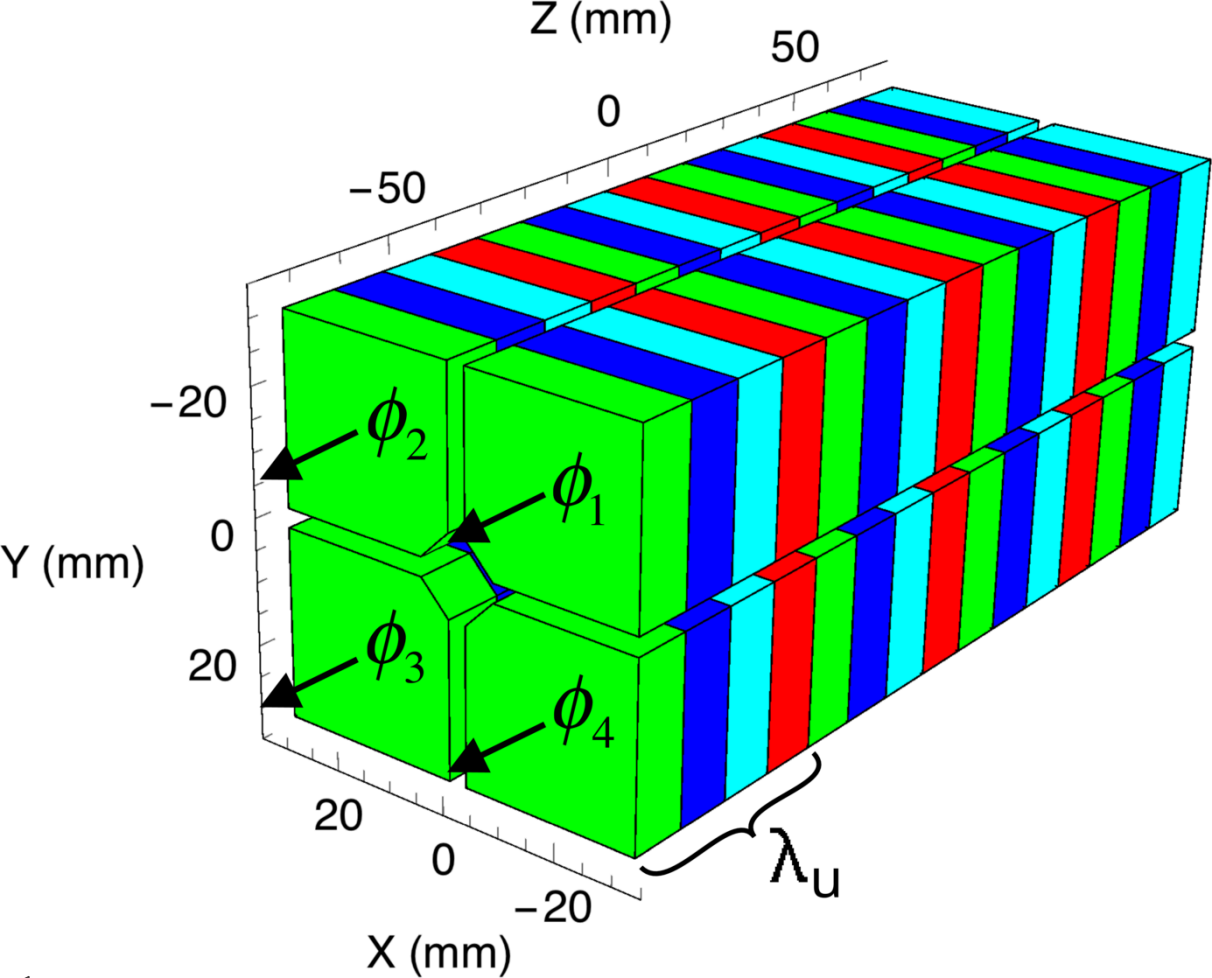
The magnetic model of an Apple X generated by the *RADIA* code (Chubar *et al.*, 1998 ▸). The longitudinal position of each row is represented by the variable ϕ_*n*_ = 0, at its initial position (before the change of variable) which generates an identical zero field on-axis. There are four different directions of magnetization, represented by four colors, where one set forms a single undulator period λ_u_.

Apple X are the most recent evolution of the advanced polarization planar light emitter (Apple) undulators (Schmidt & Calvi, 2018 ▸). As is the case with all predecessors, they consist of four magnetic rows of permanent magnets assembled following the Halbach configuration (Halbach, 1983 ▸). Each magnet has the same geometry, but two different magnetization directions exist: one follows the beam axis (type A) and the other its perpendicular plane (type B), also called the transversal plane. Specific to the Apple X, the type B are magnetized at 45°, in the plane along the line which crosses the magnetic center. Both magnet types come with positive or negative polarity and a period (λ_u_) is composed of four magnets, as schematically presented in Fig. 1 ▸.

The Apple X undulator is equipped with eight motors (eight rotational and eight absolute linear encoders) to control independently the radial and longitudinal positions of the four rows, as well as their radial distance from the magnetic center. This radial motion is the main difference with respect to the Apple III undulators, which consist of the same magnetic arrays, but can only be displaced upwards and downwards with a regular gap drive system. Thus the type B magnets of an Apple III can point towards the center only at one gap (if at all), while in the Apple X this is the case for any configuration. This characteristic allows the same photon energy to be reached for any polarization, equivalent to an exchange of coordinates *x*′ = *y* and *y*′ = *x*. In other words, it is always possible to change the helicity or to rotate the polarization by 90° for any photon energy.

There are two main operation modes to change the polarization: one is called the parallel (P) mode and produces elliptical polarizations; the second is the anti-parallel (AP) mode and produces linear polarizations with an arbitrary angle, α. In the P mode opposite rows 1 and 3 are moved against 2 and 4 by the same amount in the *z*-axis. By convention, when the four rows are at the zero shift position, the undulator produces linear horizontal polarized (LH) light: the electric field of the light is parallel to the *x*-axis. When the rows are translated in the described fashion, the light assumes a certain degree of circular polarization (C) which increases to 100% when the shift is λ_u_/4 (or 90°) and transforms to pure linear vertically (LV) polarized light at λ_u_/2. Similarly, for a parallel shift in the opposite direction, the polarization changes from LH to C to LV but with opposite helicity: at −λ_u_/4 the circular polarization is left handed, while at +λ_u_/4 it is right handed. At −λ_u_/2 the light is again LV polarized with no difference with respect to its symmetric configuration. In the AP mode rows 1 and 3 are moved against each other, while 2 and 4 are static. This results in linear polarization angles (α) between 0 and 90°. To cover the remaining angles (90° to 180°), rows 2 and 4 are moved against each other, while 1 and 3 remain at zero shift. This range α ∈ (0, 180°) can be covered with only four independent translational degrees of freedom.

## 3. Dependence of Stokes parameters on undulator magnetic field

**Figure 2:**
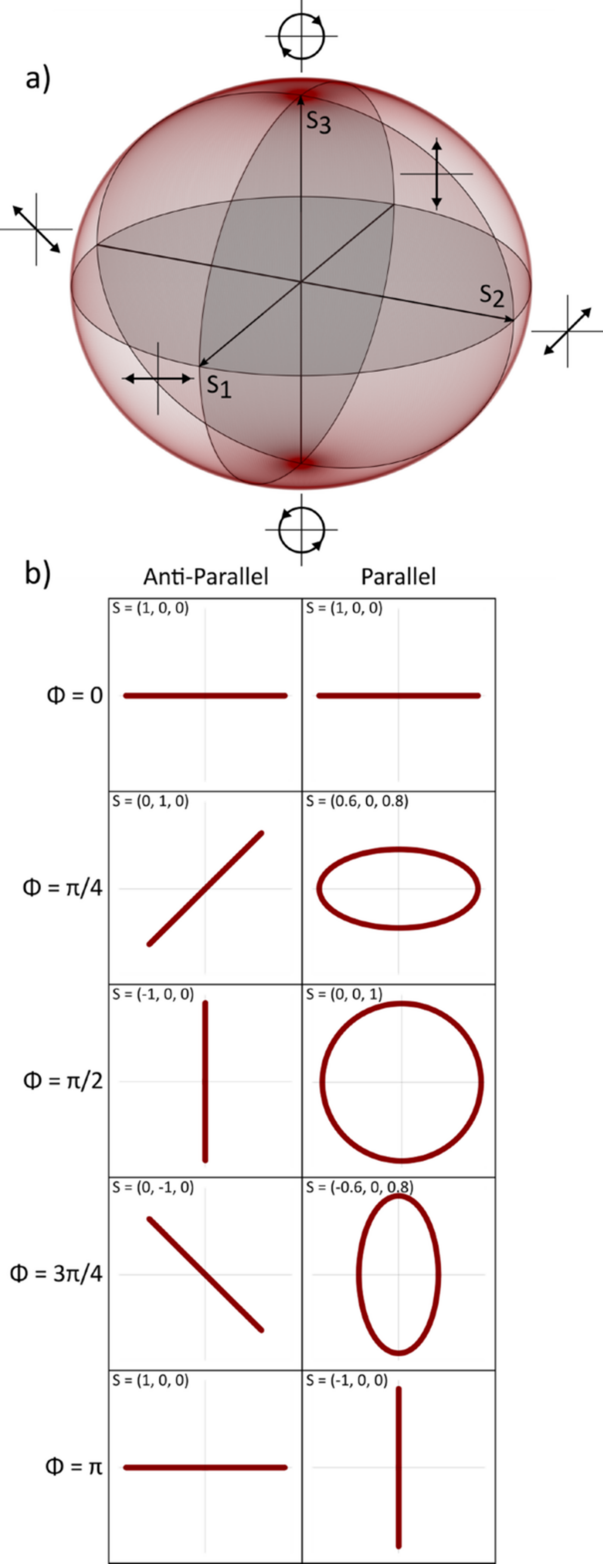
(*a*) Graphic of the Poincaré sphere on which the Stokes vector points are shown. Each point on the Poincaré sphere represents a polarization state with specific orientation, ellipticity and helicity. (*b*) A visualization of the polarization state corresponding to a set of Stokes vectors achievable by translating the Apple X (undulator phase ϕ) in anti-parallel (column 1) and parallel mode (column 2).

The polarization state of a beam of light is in general described by a set of three parameters (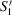, 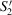, 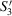) called the normalized Stokes parameters (*i.e.*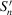 = 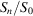). For fully polarized light, these represent a normalized vector (denoted by primes) on the Poincaré sphere [Fig. 2 ▸(*a*)], with vectors on the horizontal plane (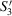 = 0) corresponding to linearly polarized light of varying orientation and vectors having a vertical component (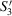 ≠ 0) possessing some degree of ellipticity. Since the Stokes vector is normalized, it is sufficient to know only two parameters to describe the shape and orientation of the polarization state, with the sign of the last describing the helicity of elliptical or circular polarizations. A visualization of the polarization state for a selection of Stokes parameters is presented in Fig. 2 ▸(*b*). Translation of the undulator magnet arrays in the P and AP operation modes effectively correspond to a rotation of the Stokes vector around the vertical and horizontal planes, respectively. It is important to note that the sign of the 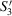 parameter is set by convention, depending on whether one is looking towards or away from the direction of beam propagation.

### 3.1. Undulator magnetic model

The magnetic field of an Apple type undulator can be expressed as the sum of the field generated by each of its four magnetic rows (Calvi *et al.*, 2017 ▸). Limiting the investigation to the on-axis transversal field (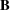 = 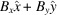), this statement can be summarized through the following equation, 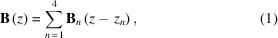
where *z* is the spatial coordinate along the magnetic axis and the index *n* runs over the four magnetic rows. Equation (1) can be expressed as a function of one of the four rows, 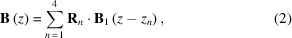
using the axis symmetries represented by the matrix **R**_*n*_, 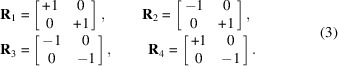
Equation (2) has a simpler formulation in the Fourier domain (denoted with a hat, ‘ 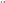
’): 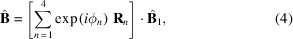
where the shifts in the *z*-axis are now substituted by four complex numbers with phase ϕ_*n*_ = 2π*z*_*n*_/λ_u_ known as the undulator phase and 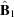 is the on-axis magnetic field generated by one of the magnetic rows (by convention, the first quadrant, labeled with the subscript ‘1’, see Fig. 1 ▸). Equation (4) can be conveniently expressed in the following matrix form,
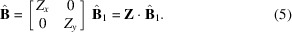
The effective deflection parameter to be used in the fundamental undulator equation, hereafter 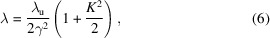
is given by 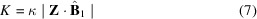
and its components (*x* or *y*) 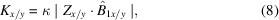
where 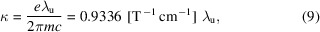
and γ is the Lorentz factor. This mathematical formulation is very general and can describe any magnetic configuration on-axis, reachable by an arbitrary shift of the four rows. Following the approximation presented by Walker (1998 ▸) which holds for the first harmonic and small *K*, it is possible to calculate the Stokes parameters directly via the magnetic field components assuming that *E*_*x*/*y*_ ≃ *B*_*y*/*x*_, using the formulas below, 
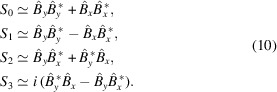
Before proceeding, it is convenient to apply the following change of variable, 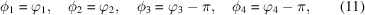
to be consistent with the regular convention that φ_1_ = φ_2_ = φ_3_ = φ_4_ = 0 corresponds to linear horizontal polarization.

### 3.2. Parallel mode or elliptical polarization

**Figure 3:**
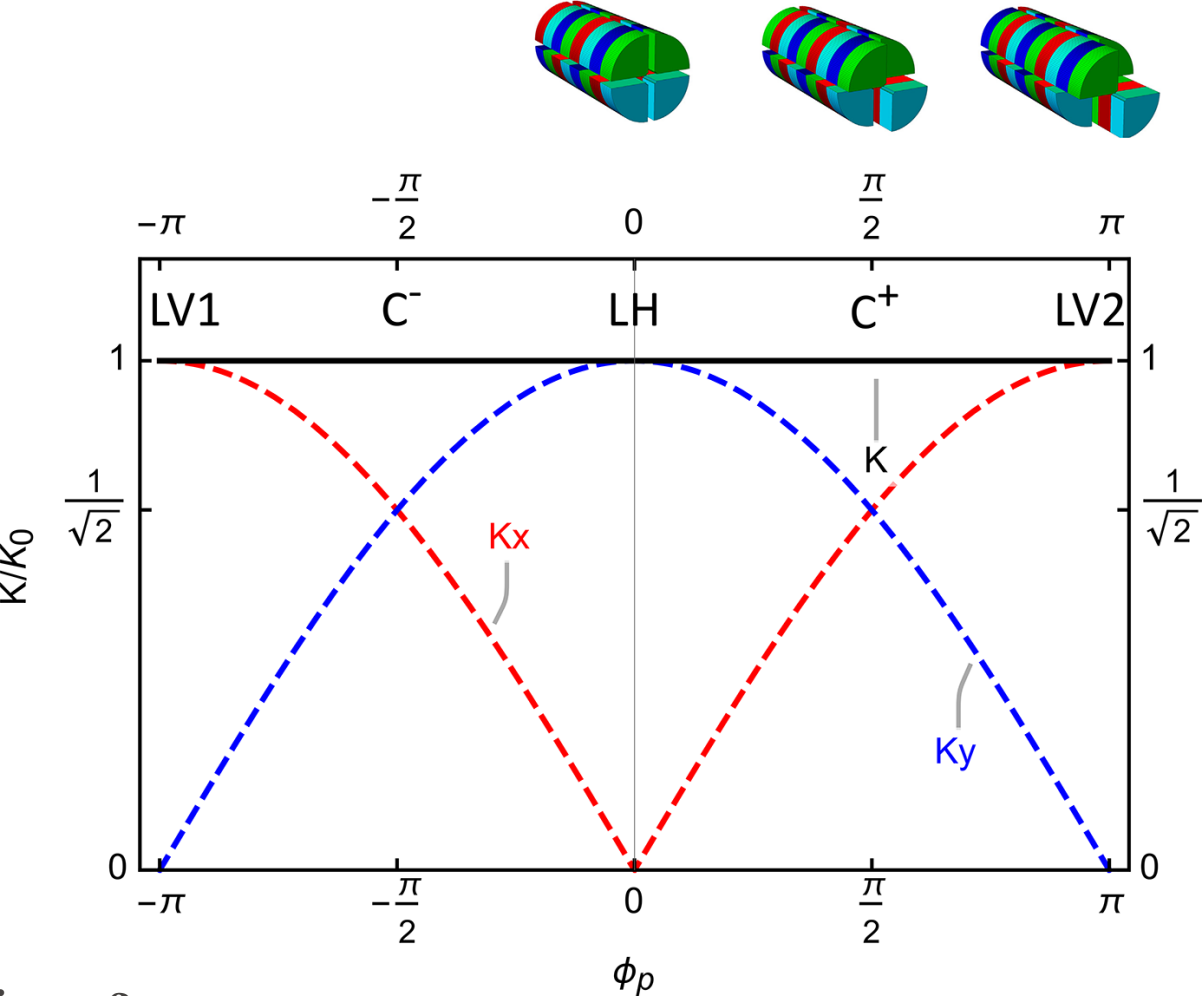
The model represents the *K* value of an ideal Apple X as a function of the parallel shift, ϕ_p_. It indicates a constant photon energy at different polarization which is only partially true for a real device.

In parallel mode, the normalized distance ϕ_p_ between rows 1 and 3 and rows 2 and 4 controls the degree of elliptical polarization of the light and can be expressed by the following relation, 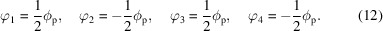
Solving equation (8) using the above assumption gives the expression 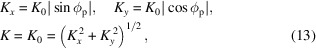
and depicted in Fig. 3 ▸, where 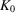 = 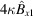, which indicates that in the ideal case an Apple X operated in parallel mode has always the same *K* and consequently the same photon energy. Using equation (4), the normalized Stokes parameters have the following values as a function of the parallel shift ,
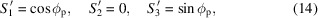
where the second term is identically zero.

In a real device, *K* is not a constant value, as was indicated in equation (13), but actually deviates by a few percent (Liang *et al.*, 2021 ▸). Thus the actual control model incorporates systematic, as well as some empirical, parts to compensate for the deviations of each undulator module.

### 3.3. Anti-parallel mode or linear inclined polarization

**Figure 4:**
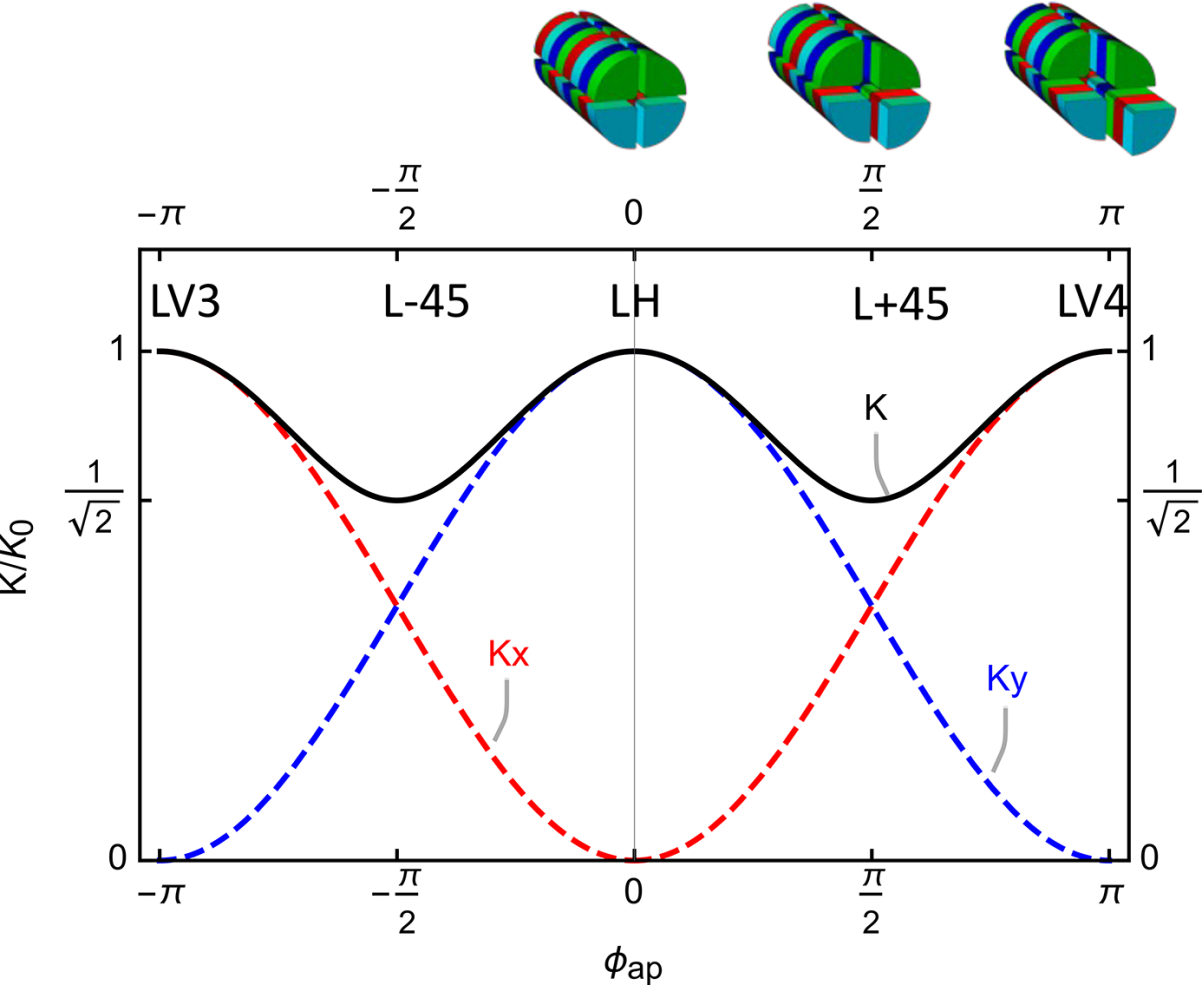
The model represents the *K* value of an ideal Apple X as a function of the anti-parallel shift, ϕ_ap_. The minimum *K* is around the angle of ±45°, corresponding to a shift of ±π/2.

In antiparallel mode, either rows 1 & 3 or rows 2 & 4 are moved against each other while the other remains, respectively, at zero position to cover the full range of linear polarizations from 0 to 180°. The explicit dependence for the first case is formulated in the following, 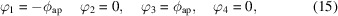
and by convention we assume that positive ϕ_ap_ corresponds to a shift of 1 and 3 with a positive α, while negative ϕ_ap_ to 2 and 4 with a negative α. With these assumptions, the deflection parameter reads like 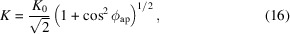
associated with the following polarization angle, 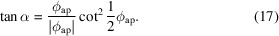
In Fig. 4 ▸, equation (16) is represented for clarity. The normalized Stokes parameters as a function of ϕ_ap_ have the following expressions, 
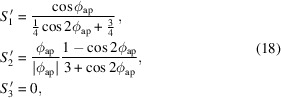
where in this case the third term is identically zero. Similar to the previous case of elliptical polarization, the model has to be empirically improved to accurately predict the radiation wavelength during operation.

## 4. Polarization measurement using recoil ion momentum spectroscopy

The polarization of the soft X-ray pulses was determined using cold target recoil ion momentum spectroscopy (COLTRIMS) (Dörner *et al.*, 2000 ▸). The technique is based on reconstructing the three-dimensional photoelectron emission patterns from the well understood photoionization process in helium atoms with polarized light. Here, the angular electron emission pattern is determined indirectly by detecting their mirror image, the momentum distribution of the photoions. Due to momentum conservation, the momentum imparted by an emitted photoelectron is transferred in full to the photoion. Thus, by measuring the three-dimensional momenta of the recoil ions, we are able to reconstruct the three-dimensional emission patterns of the photoelectrons and in turn the X-ray polarization. The measurement is performed with the so-called Reaction Microscope (ReMi) or COLTRIMS spectrometer at the Maloja endstation at SwissFEL.

Here, we describe the basics of recoil ion momentum spectroscopy, the expected photoion/photoelectron emission patterns, the experimental setup and the analysis of the polarization measurements.

### 4.1. Recoil ion momentum spectroscopy

Photoionization of an isolated atom with an X-ray photon, resulting in the emission of a single electron and a (recoil) ion, is covered by the following equations of momentum conservation, 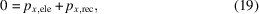

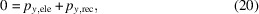

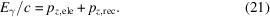
Here, we assume that the X-ray photons with energy *E*_γ_ propagate in the *z*-direction and we denote the momenta and masses of the photoelectron and the recoil ion with *p*_ele_ and *m*_ele_, and *p*_rec_ and *m*_rec_, respectively. The electron’s binding energy is *E*_bind_. Combining momentum and energy conservation, under the assumption that the resulting ion after photoionization stays in its electronic ground state and neglecting the momentum of the photons, yields an expression which describes a sphere in momentum space with a constant radius (Dörner *et al.*, 2000 ▸),
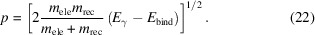
Thus, the products of photoionization (photoelectrons and recoil ions) are ejected with the same absolute momenta *p* in opposite directions. The emission pattern of the electron is mirrored in the emission pattern of the recoil ion. Due to the mass ratio of 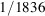 between an electron and a proton, most of the energy is carried by the photoelectron.

### 4.2. Photoionization with soft X-rays

**Figure 5:**
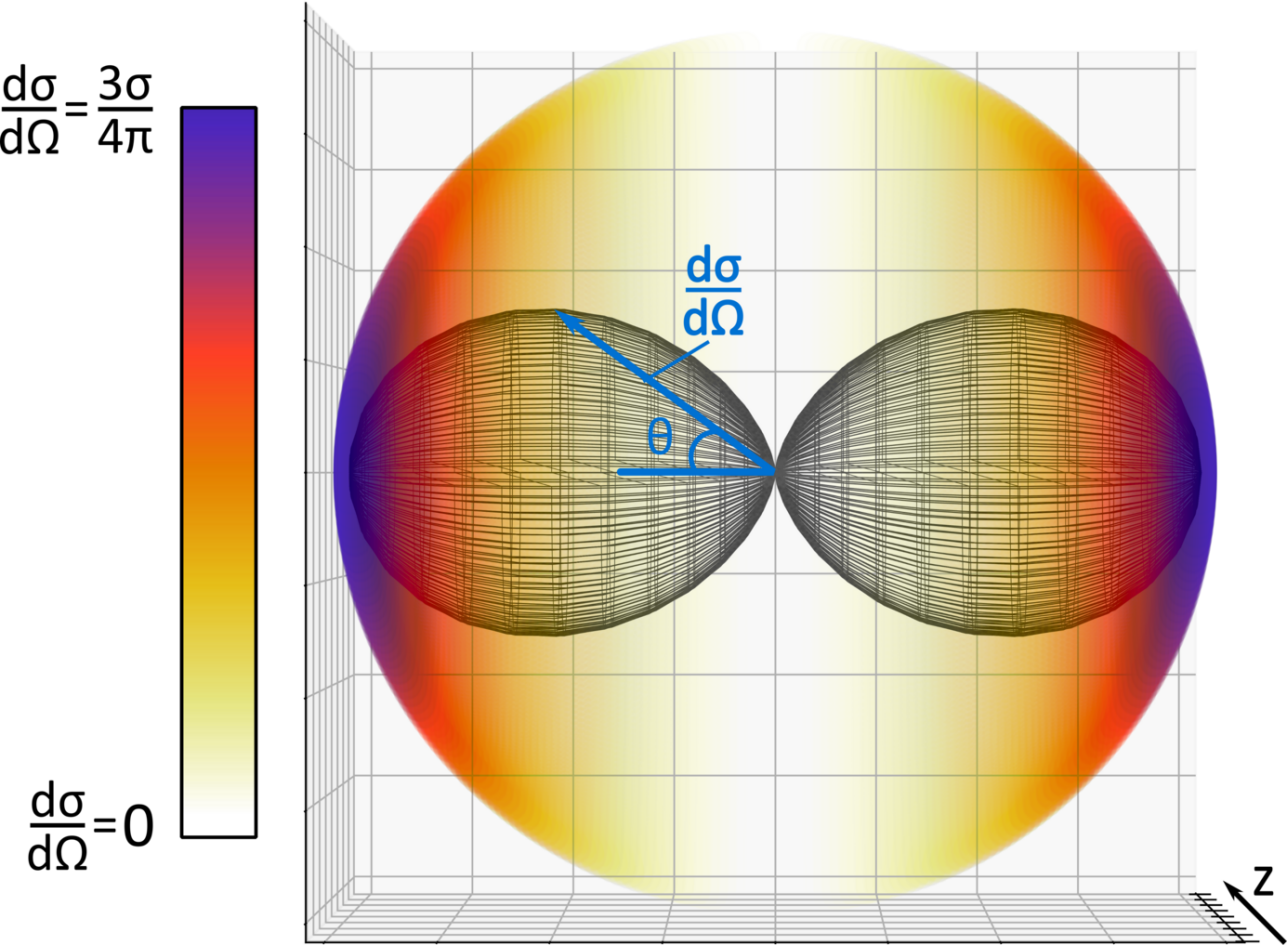
Expected angular distribution of photoelectrons emitted from neutral helium upon absorption of a single photon from a linear polarized beam, as described by equation (23). The black surface mesh shows the magnitude of the cross section (radial distance) versus the ejection angle relative to the polarization axis (θ, ψ) while the colored sphere represents the angular distribution of photoelectrons expected from the measurement for single photon ionization of helium with a linearly polarized beam.

In photoionization, the angular distribution of the emitted photoelectrons from a certain atomic subshell is described by (Schmidt, 1992 ▸) 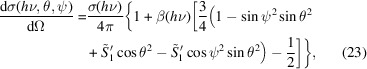
where *h*ν is the photon energy, σ(*h*ν) the partial cross section for a certain photon energy, θ the angle between the electric field vector of the X-rays and the ejected electron, ψ the azimuthal angle of the ejected electron around the horizontal plane, 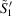 the reduced Stokes parameter which describes the excess of linear polarization and β(*h*ν) the photon-energy dependent asymmetry parameter. Since our measurement is performed for a fixed photon energy, the cross section and the β parameter are constants. Thus, for a fixed photon energy and in the polarization plane (ψ = 0), equation (23) simplifies to 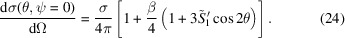
Therefore, the photoelectron ejection characteristic can be described by β if the cross section σ is known. In the soft X-ray regime, the single photoionization process in the most simple neutral rare-gas atom, helium with its two 1*s* electrons, is well understood and characterized by β = 2 (Fig. 5 ▸). Thus, the measured photoelectron or recoil ion angular distribution can inversely be used to determine the angle and degree of linear polarization of the photon beam.

### 4.3. Reaction Microscope (ReMi)/COLTRIMS

**Figure 6:**
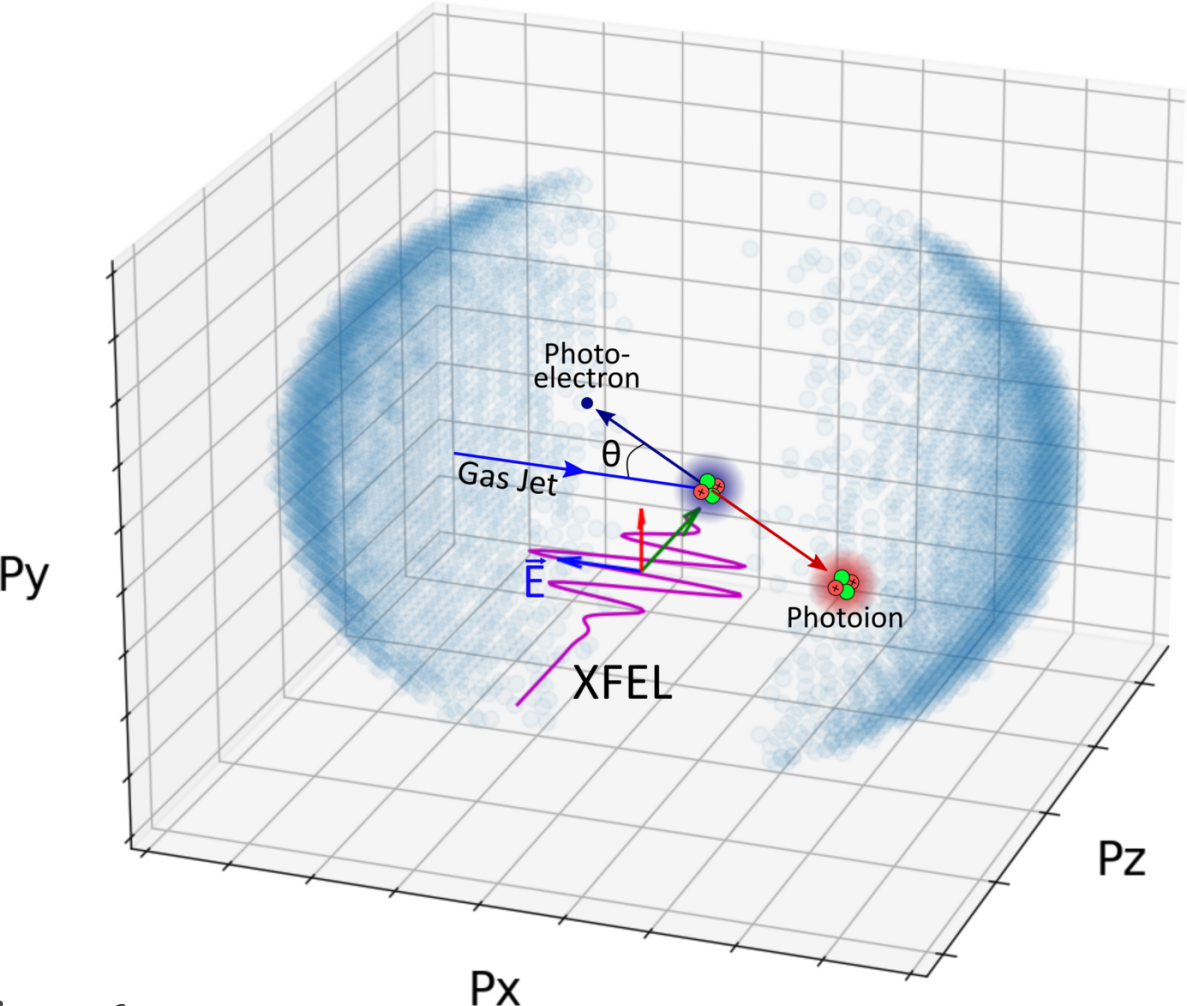
Photoionization scheme used to measure the XFEL polarization. Helium atoms from a pulsed gas jet are photoionized by absorption of a single X-ray photon of linear horizontal polarization and a photoelectron is ejected while the photoion recoils with opposite momentum. The photoion momenta are measured in all directions using COLTRIMS, resulting in the 3D momentum distributions (blue circles) when summed over several thousand events.

We briefly summarize the key characteristics of our ReMi/COLTRIMS; for details on the operation principle we refer to Dörner *et al.* (2000 ▸) and Ullrich *et al.* (2003 ▸). A cold atomic beam of helium atoms is generated using a 100 Hz pulsed Even–Lavie valve (Even, 2014 ▸) with a backing pressure of 10 bar in combination with two skimmers with 1 mm diameter each. Given the comparably small ion recoil imparted by the electron, the atomic beam must be sufficiently cold in order to avoid thermal motion smearing out the reconstructed ion momenta. Based on the expansion parameters for He (nozzle diameter = 150 µm), we estimate a gas temperature below 1 K (Scoles, 1988 ▸). The gas jet is mounted horizontally in the *x*-direction and the electric field of the ion spectrometer is oriented vertically in the *y*-direction at a 90° angle to the X-ray propagation direction and to the gas jet (*cf*. Fig. 6 ▸).

Singly charged helium ions, generated upon single photoionization with the soft X-rays, are accelerated in a homogeneous electric field of 0.83 V cm^−1^ over a distance of 6 cm. No drift region is implemented. Using a 80 mm-diameter hexagonal MCP-delay-line detector (Jagutzki *et al.*, 2002 ▸), the impact time and position are determined, which allows the three-dimensional momentum of each detected ion to be calculated.

**Figure 7:**
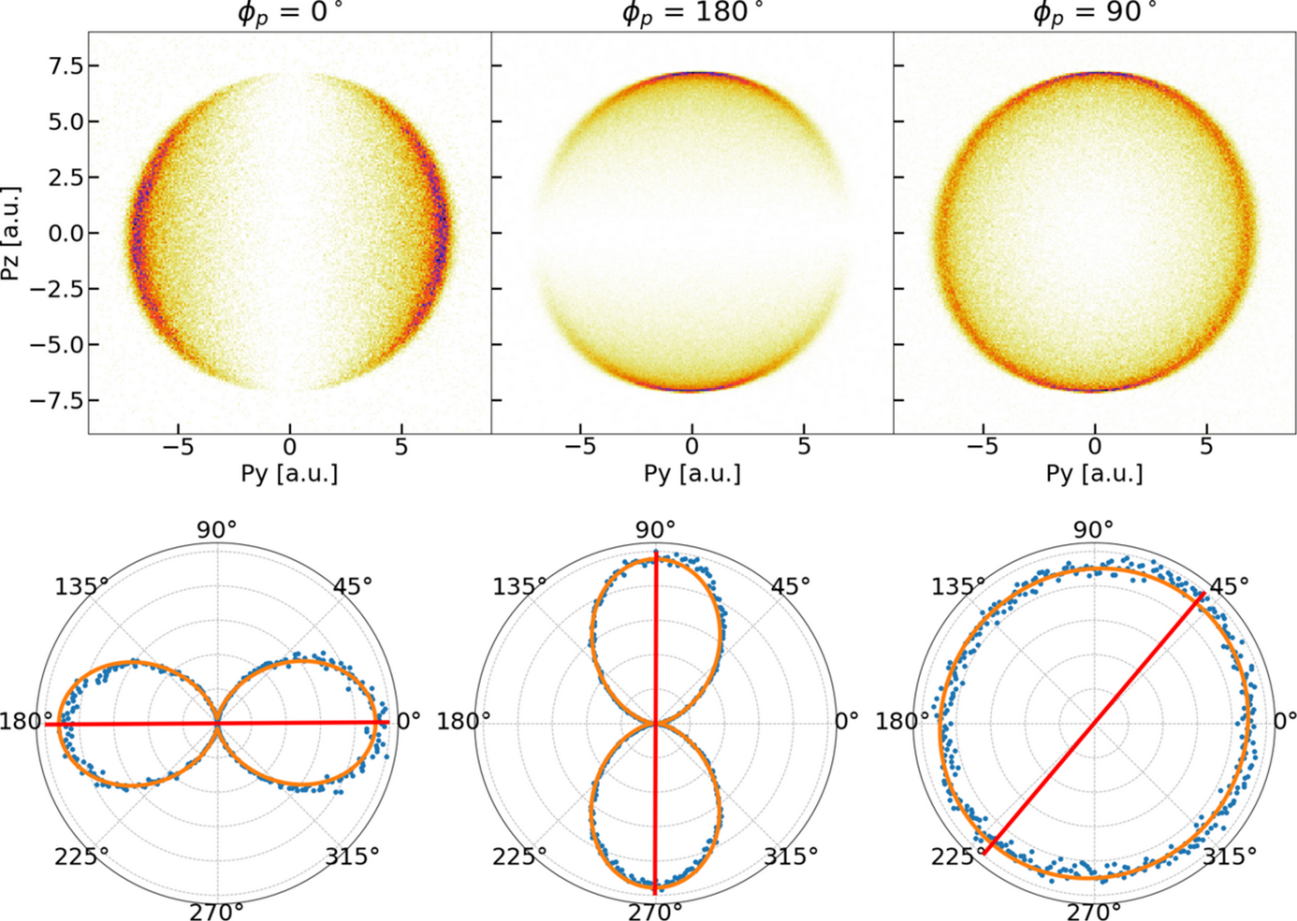
Raw photoion momentum distributions for linear horizontal (top left), linear vertical (top center) and circular polarizations (top right). The momentum resolution along the time-of-flight (vertical) axis is better than along the position detection (horizontal) axis. The circular polarization results in a circle with a radius of 7 a.u. and is used as a reference to assess the quality of the calibration. The bottom panels show for each of these polarizations the momentum distributions integrated over the radial coordinate (blue dots), their corresponding fit (orange curve) and the retrieved polarization angle (red line).

The momentum calibration for a recoil ion with charge *Q* and mass *m* is based on the physical properties of the experimental setup, specifically the detector geometry, the spectrometer length and the applied voltages. According to equation (22), the recoil ion signal for a fixed photon energy shall be (in first approximation) on a sphere with radius 7 a.u. (atomic units) for singly ionized He which has a binding energy of 24.6 eV. Using circularly polarized light (*cf*. Fig. 7 ▸, top right), which should result in a circle in the *xy*-plane when averaging over many ionization events, we can assess the quality of the calibration. Note that the momentum resolution in the *y*-direction is better than in the *x*-direction due to the higher resolution that can be achieved in the time-of-flight (TOF) measurement as compared with the spatial resolution from the delay-line detector. This manifests as a narrower momentum distribution in the *y*-direction.

For the calibration, we used a fixed photon energy of 700 eV. The polarization angles for all measurements have been calibrated using the nominal setting for linear vertical polarization. The calibration datasets contained approximately 3 × 10^5^ counts while the linear and elliptical polarization datasets contained 1.3 × 10^5^ and 2.3 × 10^5^ on average, respectively.

### 4.4. Polarization analysis

The recoil ion momentum maps can be analyzed to extract the angle and degree of linear polarization of the FEL beam. The 3D momentum distributions were first projected along the propagation (*p*_*z*_) coordinate, yielding 2D recoil ion distributions in *p*_*x*_ and *p*_*y*_, which are demonstrated in Fig. 7 ▸ (top row) for three basic polarization cases (LH, LV, 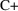). As noted above, the difference in momentum resolution along the two orthogonal axes is apparent. However, this does not change the total number of counts detected along each axis, and so does not strongly affect the integrated distributions. These histograms were then converted to polar coordinates and the counts were integrated over the radial coordinate, providing distributions of total radial counts as a function of the polar angle.

The resulting angular distributions were analyzed by fitting to 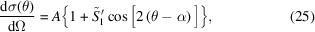
derived from equation (24), where *A* is a free scaling parameter, α is the polarization angle and 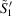 = 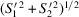 is the reduced Stokes parameter which is obtained directly from the fit. From this, 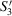 = 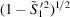 is calculated assuming a fully polarized beam. This assumption is necessary as 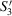 has no direct contribution to the photoionization cross section and can only be estimated in this experimental scheme using the residual of the 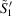 component (Huang, 1980 ▸). For this reason, it is impossible to distinguish between circularly polarized and unpolarized components of the angular cross section without measuring XCD signals. Stokes parameters of 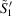 = 1 and 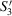 = 0 correspond to linearly polarized light, 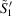 = 0 and 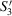 = ±1 correspond to circular polarized light and elliptical polarizations are characterized by Stokes parameters in between. From these and the polarization angle α extracted from the fit, the Stokes parameters 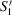, 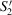 and 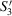 are obtained.

The bottom plots in Fig. 7 ▸ visualize the projections of the raw photoion momentum distributions and their radial integrations in polar coordinates for the aforementioned basic polarization cases.

Using a projection of the entire sphere along the propagation direction is useful for precisely determining the polarization angle, but cannot be used to accurately determine the reduced Stokes parameter as the angular cross section has a dependence on the azimuthal angle ψ and this information is lost in the projection. Thus, to determine 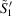, a thin (±0.5 a.u.) slice of the 3D momentum distribution in the plane perpendicular to the direction of propagation at ψ ≃ 0 is used for fitting to equation (25) instead of the projection of the entire sphere. Using slices has the added benefit that background counts that exist outside the ring at *p* = 7 a.u. can be excluded by integrating only over the ring.

## 5. Results and discussion

**Figure 8:**
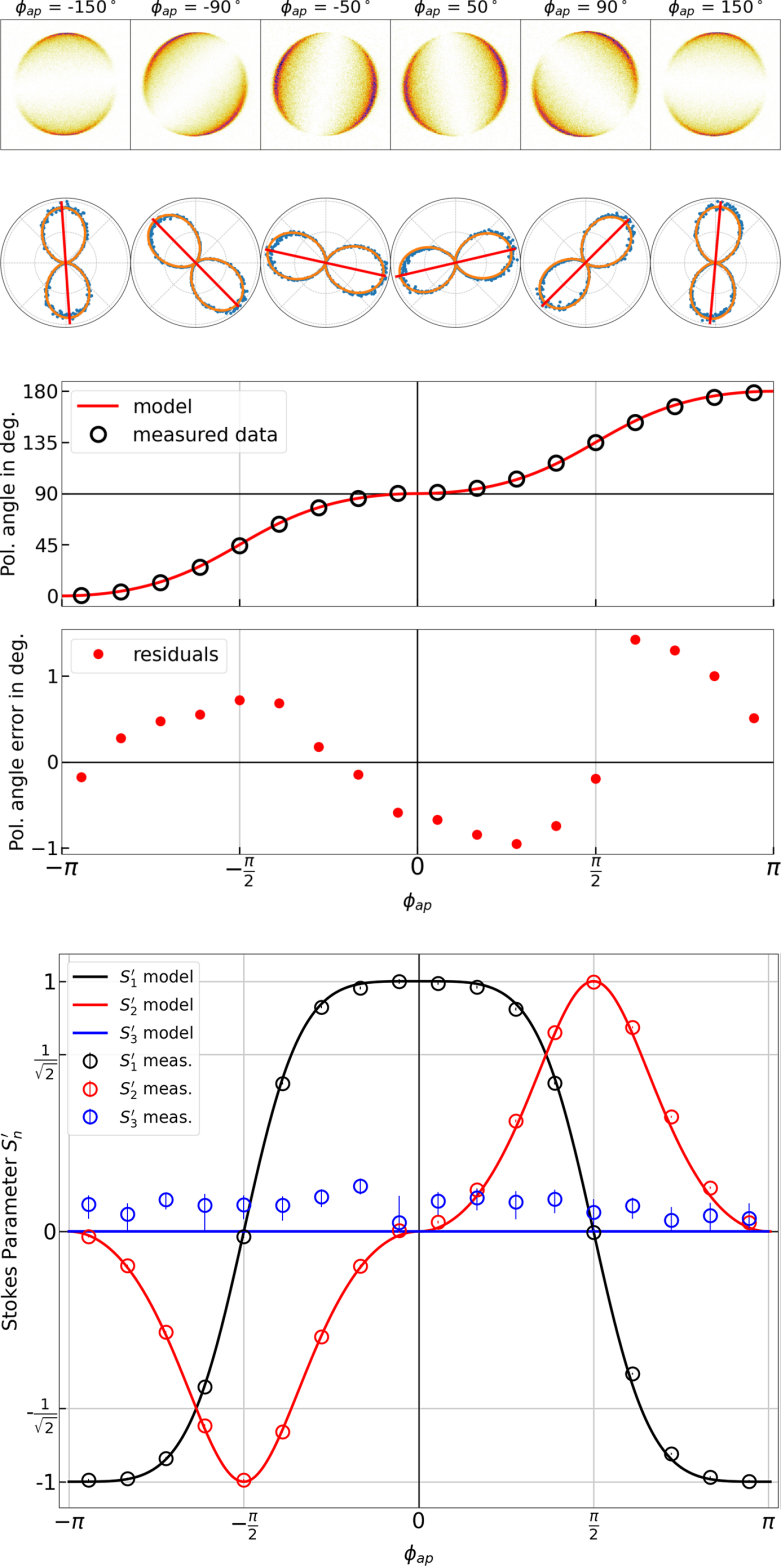
Top: raw photoion momentum distributions, integrated projections (blue dots), fits (orange lines) and retrieved polarization angles (red lines) for linear polarizations generated at various anti-parallel undulator phases ϕ_ap_, showing examples over the range of undulator phases. Center: model for the polarization angle in the linear inclined mode (red line) overlaid with the measured values (circles) with residuals below. Bottom: model for the Stokes parameters in the linear inclined mode (solid lines) overlaid with the measured values (circles).

We measured various linear inclined (antiparallel mode) and elliptical (parallel mode) polarizations. The photoion momentum distributions and fits for some linear polarizations are shown in Fig. 8 ▸ (top). The distributions are well described by equation (25) as shown by the quality of the fits. In the center part of Fig. 8 ▸, the polarization angles extracted from the fits are overlaid with the model as described by equation (17). The measured polarization angles agree well with the model, with a root mean square deviation of 0.74°. The errors shown below appear to be systematic, being larger for tilted than for horizontal or vertical polarizations, and of the same order as the estimated experimental uncertainty of 0.5° based on the fit and calibration errors. One potential reason for the observed steps in the residuals might be a magneto-mechanical hysteresis, *i.e.* a slight movement of the magnets, due to high magnetic forces at certain undulator phases combined with a mechanical instability, similar to what was discovered at the European XFEL (Karabekyan *et al.*, 2022 ▸). A reduced Stokes parameter of 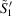 = 0.994 ± 0.004 was obtained on average over the measured range of linear polarizations in the anti-parallel mode. The bottom of Fig. 8 ▸ shows the Stokes parameter calculated for all linear inclined measurement points as well as its corresponding model for the anti-parallel mode of the undulator, where a good agreement is observed.

**Figure 9:**
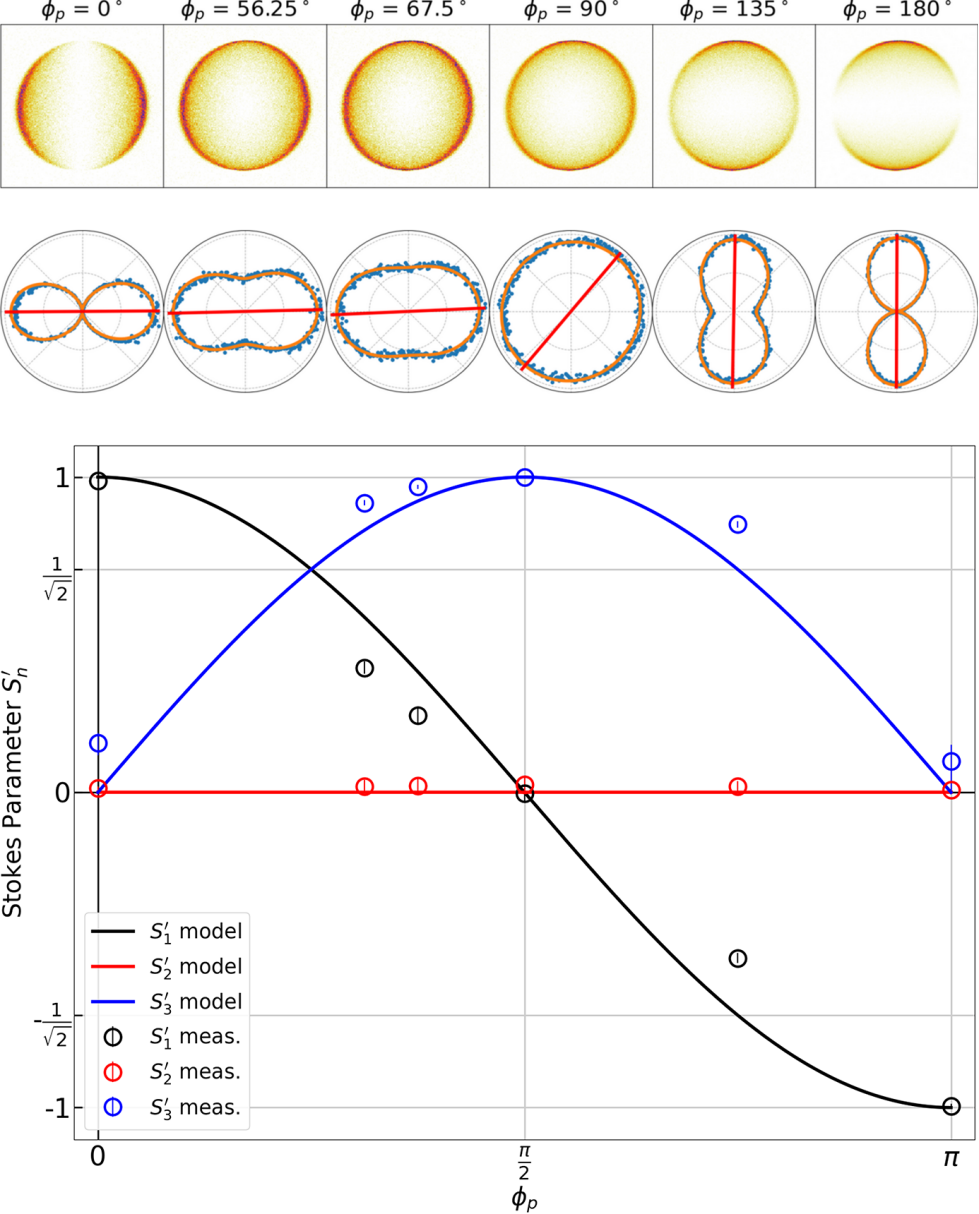
Top: raw photoion momentum distributions, integrated projections (blue dots) and fits (orange lines) for elliptical polarizations generated at various parallel undulator phases ϕ_p_, showing the transition from linear to elliptical to circular polarization. Bottom: measured Stokes parameters (circles) as a function of the undulator phase overlaid with the model (solid lines) from equation (14).

The top of Fig. 9 ▸ shows data for elliptical polarizations at various undulator phases ϕ_p_, with their corresponding analysis below. Elliptical and circular polarizations were characterized in the same way by fitting the sliced distributions to equation (25) without changing fit parameters. As the diagonal magnet arrays of the undulators are translated in the parallel mode, the polarization shifts from linear horizontal (at ϕ_p_ = 0), to elliptical horizontal, to circular (at ϕ_p_ = π/2), to elliptical vertical, to linear vertical (at ϕ_p_ = π).

It is important to note that this momentum spectroscopy measurement cannot distinguish between left-hand or right-hand helicities for circular and elliptically polarized light, nor between circularly polarized and unpolarized light as these would result in the same constant angular distributions (Huang, 1980 ▸). To unambiguously determine the 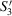 component without assumptions, it would be necessary to perform XCD measurements, as performed by Veyrinas *et al.* (2013 ▸) and Oura *et al.* (2007 ▸). However, based on magnetic measurements of the Apple X undulators (Liang *et al.*, 2019 ▸; Kittel *et al.*, 2019 ▸) it is safe to assume that the radiation produced by the undulators in the circular polarization mode is fully polarized and has the correct helicity.

The Stokes parameters should depend on ϕ_p_ as described in equation (14). The bottom of Fig. 9 ▸ shows the measured Stokes parameters as a function of the undulator phase overlaid with equation (14). Here the undulator phases corresponding to LH, LV and C polarizations agree well with the model, but the measured Stokes parameters for the elliptical polarizations exhibit a lesser 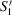 component than expected from the model, and appear to follow more of a linear dependence on the undulator phase rather than the expected cosine dependence. One potential source of disagreement could be a difference in beamline transmission along horizontal and vertical directions stemming from the three reflections (two horizontal and one vertical) present in the beam path. However, this difference is expected to be small due to the grazing incidence of the mirrors. Additionally, this would translate as a measurable ellipticity in the circular polarization mode, where no appreciable ellipticity is observed. Another possibility is that an unpolarized component is present in the elliptical polarizations which cannot be distinguished from the circularly polarized component. Additional numerical modeling and measurements directly sensitive to 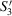 are required to determine the source of the deviations for the elliptical polarizations. Nonetheless, Fig. 9 ▸ demonstrates that the ellipticity of the polarization state can be effectively tuned in the parallel operation mode.

The results of the undulator phase scans in parallel and antiparallel modes show that both the angle of linear polarization and the degree of circular polarization can be precisely controlled to fulfill the vast majority of requested polarization modes for users of the Athos beamline.

The main advantages of a COLTRIMS setup over a circular array of electron spectrometers in relation to polarization metrology lie in the greater angular sampling and self-referencing nature of the detection method. With a COLTRIMS setup, it is possible to measure photoelectron angular distributions with much finer angular sampling (2° in this work versus 22.5° with 16 TOF spectrometers) over a 4π solid angle using a single detector. Using a single detector is an advantage as each TOF spectrometer in an array must be precisely calibrated to have equal sensitivity in order to avoid errors in the retrieved polarization state. On the other hand, COLTRIMS poses several experimental challenges. Such measurements, which are more sensitive to experimental conditions than TOF arrays, must be performed in vacuum conditions approaching 10^−11^ mbar and have a complex momentum calibration procedure. Additionally, COLTRIMS measurements rely on low count rates (typically <10 counts per X-ray pulse) and thus cannot be used for single-shot characterization of the polarization state, unlike TOF arrays. For comparison, a single polarization dataset containing 100000 counts requires approximately 20000 X-ray pulses, equivalent to 200 s of continuous acquisition at the 100 Hz repetition rate of Athos. For these reasons, we envision that reaction microscopes, which are relatively commonplace at XFEL facilities, could be useful for ‘one-time’ precise characterizations of the polarization output of an undulator line where a high degree of angular sensitivity is required, rather than as dedicated tools for routine and single-shot polarization characterization where TOF arrays would be better suited.

## 6. Conclusion

We have demonstrated the generation and characterization of linear inclined and variable elliptical polarizations at SwissFEL’s soft X-ray beamline Athos by measuring the polarization state as a function of the undulator phase in both parallel and antiparallel operation modes. To this end, we derived two separate functional models to control the polarization in Apple X undulator modules in order to fulfill any Athos user requests. We used cold target recoil ion momentum spectroscopy to determine the X-ray polarization, making use of the well known photoelectron emission pattern in He.

Our characterization results for polarizations in the antiparallel mode verify our model on the order of half a percent, which is on the same order as the error we expect from the measurement. Our characterization of polarizations in the parallel mode revealed systematic deviations from the expected model for elliptical polarizations, the origin of which cannot be determined in the present study and will require further XCD measurements to directly measure the circular polarization component without assumptions on the overall degree of polarization.

In short, Athos, an XFEL undulator line consisting only of Apple X modules, enables an unprecedented degree of flexibility as well as a clean polarization control in a compact form. This degree of control allows new types of polarization-sensitive measurements, currently unique to Athos, such as independently polarized two color X-ray-pump/X-ray-probe experiments.
